# Medical Costs of Stroke Care Between Women With and Without Dysmenorrhea: A Population-Based Comparison

**DOI:** 10.3389/fpubh.2021.699359

**Published:** 2021-09-03

**Authors:** Ya-Wen Lin, Fung-Chang Sung, Ming-Hung Lin, Chih-Hsin Muo, Yu-Kuei Teng, Chia-Hung Kao, Ya-Ling Tzeng

**Affiliations:** ^1^School of Nursing and Graduate Institute of Nursing, China Medical University, Taichung, Taiwan; ^2^Department of Public Health, China Medical University, Taichung, Taiwan; ^3^Management Office for Health Data, China Medical University Hospital, Taichung, Taiwan; ^4^Department of Health Services Administration, China Medical University, Taichung, Taiwan; ^5^Department of Food Nutrition and Health Biotechnology, Asia University, Taichung, Taiwan; ^6^Department of Pharmacy and Master Program, Tajen University, Pingtung, Taiwan; ^7^Department of Nursing, China Medical University Hospital, Taichung, Taiwan; ^8^Graduate Institute of Biomedical Sciences, College of Medicine, China Medical University, Taichung, Taiwan; ^9^Department of Nuclear Medicine and PET Center, China Medical University Hospital, Taichung, Taiwan; ^10^Department of Bioinformatics and Medical Engineering, Asia University, Taichung, Taiwan; ^11^Center of Augmented Intelligence in Healthcare, China Medical University Hospital, Taichung, Taiwan

**Keywords:** cost of stroke care, dysmenorrhea, menstrual cycle, type of stroke, young women

## Abstract

**Objective:** This study investigated the medical care costs of stroke type between age-matched cohorts with and without dysmenorrhea using the National Health Insurance Research Database (NHIRD).

**Methods:** We collected all 66,048 women with dysmenorrhea and 66,048 women without dysmenorrhea whose age (15-44-year-old) and index year (from 1997 to 2013) were matched for comparison. We assessed the incidence and compared the risk of stroke and stroke subtype in two cohorts. The proportional distributions of stroke subtypes by age between the two cohorts were compared among the women with stroke, and their hospitalization rate was also estimated. In addition, medical cost, length of stay, and the medical cost within 30 days after stroke were compared between the two cohorts.

**Results:** The stroke risk in dysmenorrhea was greater than comparisons (HR = 1.26, 95% CI = 1.11–1.42). Proportionally, hemorrhagic stroke (HS) significantly decreased with age in both cohorts, whereas ischemic stroke (IS) significantly increased with age when both cohorts were combined. The dysmenorrhea cohort had a higher portion of transient cerebral ischemia (TIA) stroke than comparisons (31.3 vs. 24.2%, *p* = 0.01) and a lower risk of hospitalization for IS (OR = 0.48, 95% CI = 0.21–0.69). Among the four-stroke subtypes, the cost of care for TIA was the least (US$157 ± 254). The average cost for stroke care was not significantly different between women with and without dysmenorrhea.

**Conclusion:** The hospitalization rate and medical costs of TIA are lower than other types. All women should prevent and treat TIA as soon as possible to avoid recurrence or progression to major stroke events and reduce medical costs, regardless of whether they have dysmenorrhea.

## Introduction

Dysmenorrhea and stroke are prevalent disorders in women (1–5). Dysmenorrhea, characterized by abdominal pain, cramps, nausea, headaches, and diarrhea, occurs during menstruation. Dysmenorrhea can be categorized as primary and secondary based on onset and persistence (4, 5). Primary dysmenorrhea, occurring 3 years after the first menstrual cycle, affecting young women (15–25 years of age), is the more prevalent and widely studied form of dysmenorrhea (5–8). Secondary dysmenorrhea is associated with a specific underlying cause of a reproductive organ. The global prevalence of dysmenorrhea ranges from 28 to 87.4% (4–9).

Stroke is one of the leading causes of death, after cardiovascular diseases and cancer, and is also the leading cause of disability in the United States (1). Persky and colleagues reported that each year, approximately 55,000 more women than men suffer from strokes, including recurrent strokes. If the current trend continues, it has been estimated that 60% of all stroke cases will be women by 2030 (10). In 2012, the proportional mortality associated with stroke was estimated at 7.2%, which results in an economic burden of roughly US$75 million annually. In Taiwan, stroke is the third-leading cause of death (2).

Stroke can be classified as ischemic, hemorrhagic, and transient ischemic (11). The most common stroke, referring to a blockage of blood flow to the brain, is ischemic stroke (IS), which may account for approximately 75% of all stroke cases (12, 13). Hemorrhagic stroke (HS), caused by a leakage of blood in the brain (14), has been associated with high blood pressure and aneurysms (15). Transient ischemic attack (TIA) is a warning sign of future major stroke events, such as IS. TIA is characterized by a blockage of blood flow to the brain for a short period of time, commonly <5 min (16).

Women face a high risk of stroke, and this risk appears to be growing. In Taiwan, nearly 74% of all stroke cases are IS, a much greater proportion than cases of HS (19.1%) and TIA (6.7%) (12). It should be noted that dysmenorrhea in women is associated with mutual comorbidities of stroke, including cardiovascular disorders and thyroid diseases (17–19). In the recent Chinese Project Environmental and LifEstyle FActors iN metabolic health throughout life-course Trajectories (ELEFANT), Xu et al. found that young women with dysmenorrhea are at an increased risk of hypertension, and the estimated risk increases as the duration of menstrual bleeding increases (17). A recent study using the Spontaneous Coronary Artery Dissection (SCAD) registry data of Mayo Clinic found that chest pain is prevalent in women before menstruation (18). A New Delhi study found that women with menstrual disorders are prevalent with thyroid disorders, 14% of these disorders being diagnosed with hypothyroidism and 8% of these being diagnosed with hyperthyroidism (19). Thyroid hormones play an important role in reproductive physiology, directly affecting the ovaries, and hypothyroidism has numerous effects on the cardiovascular system. Thyroid disorders are associated with IS (20).

Although stroke and dysmenorrhea are related to mutual risk factors, few researchers have investigated the association between the two conditions. A recent case–control study using insurance-claim data of women with dysmenorrhea in Taiwan suggested that stroke-related disorders are more prevalent in stroke cases than in nonstroke controls (21). The disorders included hypertension (adjusted [aOR] 4.53 [95% CI 3.46–5.92]), hyperlipidemia (aOR 1.60 [95% CI 1.19–2.15]), arrhythmia (aOR 1.80 [95% CI 1.31–2.46]), and thyroid disease (aOR1.56 [95% CI 1.20–2.02]). HS is more prevalent than IS in younger women suffering from dysmenorrhea.

Stroke can result in physical disability and dependence on others, both of which impose high economic costs (2). Stroke costs an estimated $34 billion each year in the United States (22). The pain and psychological distress caused by dysmenorrhea also prevent many individuals from performing daily activities, resulting in a loss of work hours. It should, however, be noted that it remains unclear how the occurrence of stroke among women with dysmenorrhea affects the cost of care. Few studies have evaluated whether the stroke types and costs in women with dysmenorrhea differ from those in women without dysmenorrhea (21). The occurrence of dysmenorrhea and stroke varies by age (3). In this study, we adopted a follow-up approach to conduct a retrospective cohort study to compare the occurrence of stroke and costs for care by stroke type between women with and without dysmenorrhea using insurance-claim data. The design made it possible for follow-up with the women to determine the outcomes of stroke, such as costs by the type of stroke (23).

## Materials and Methods

### Data Source

The study population was selected from the Health and Welfare Data Science Center of the Ministry of Health and Welfare, which contained the data released by the Taiwan National Health Research Institutes, documenting claims data for 1 million beneficiaries listed in the 2000 Registry of Beneficiaries. The database contained details of care provided to the insured population from 1996 to 2013, including demographic characteristics, inpatient and outpatient care, medications, relevant surgical procedure codes and examinations, and costs. Medical costs included the medical fees of the outpatient (containing diagnostic fee, treatment fee, medicine fee, and pharmaceutical service fee) and inpatient (containing diagnostic fee, ward fee, treatment fee, medicine fee, and pharmaceutical service fee). The Research Ethics Committee of the China Medical University and Hospital approved the use of insurance-claims data for this study (CMUH104-REC2-115-AR4).

### Study Cohorts With and Without Dysmenorrhea

**Figure 1:**
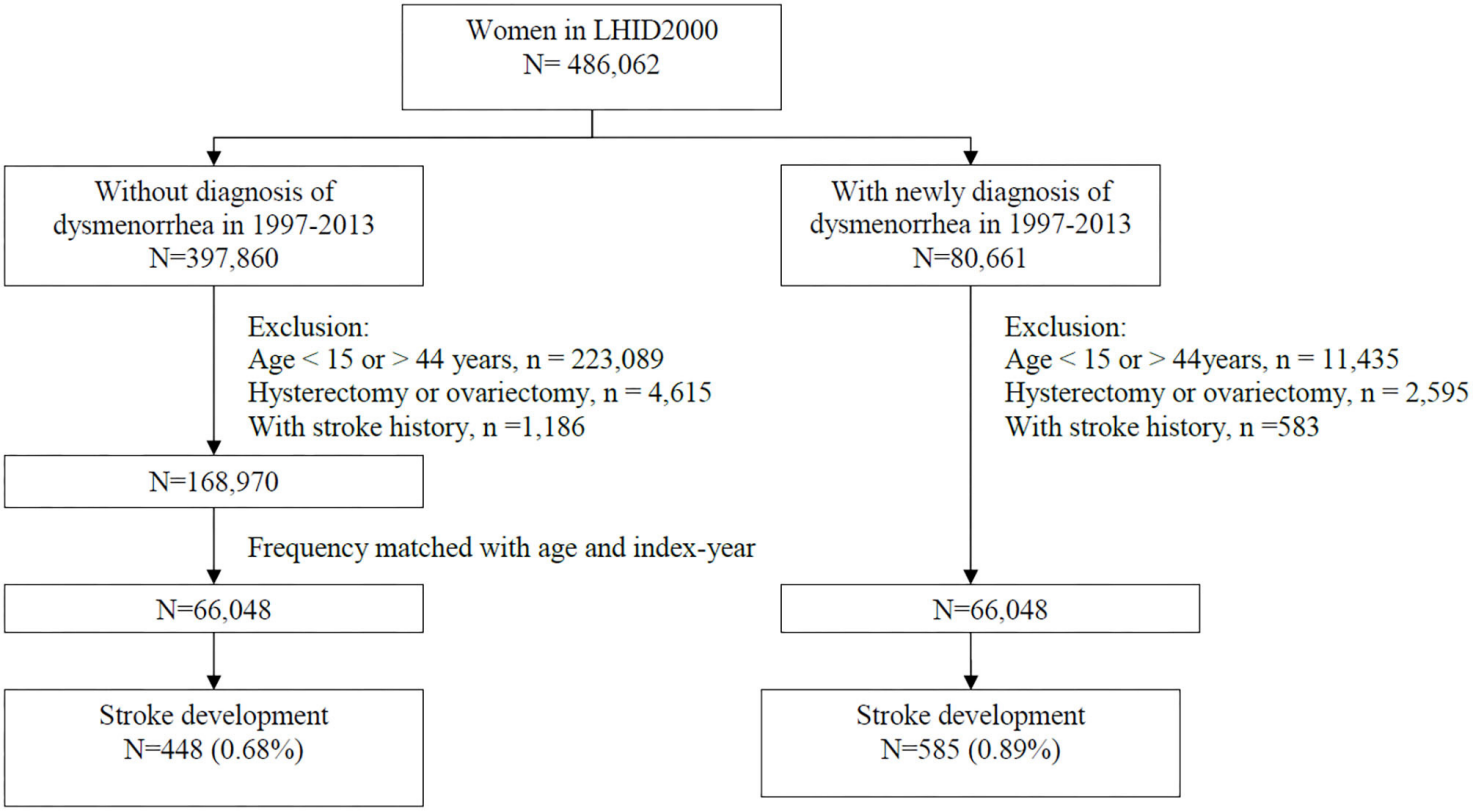
Flowchart for the process of selecting study cohorts.

From the claims data, we identified 80,661 women with diagnosed dysmenorrhea (ICD-9-CM: 625.3) for at least two times from 1997 to 2013. After excluding those aged <15 or >44 years and those with a history of hysterectomy or ovariectomy (procedure codes 68 and 65, respectively), or stroke, 66,948 women were included in the dysmenorrhea cohort (Figure 1). The dysmenorrhea diagnosed date was defined as the index date. From women without the diagnosis of dysmenorrhea, we randomly selected a comparison cohort, frequency matched by age and index year against the dysmenorrhea cohort, after excluding those with a history of hysterectomy, ovariectomy, or stroke.

### Outcome Measurements

The outcome measures included the stroke development, stroke-hospitalized rate, and the average medical cost and length of stay of stroke care.

#### Stroke Development and Stroke Hospitalization Risk

All subjects were followed up from the index date until the date of stroke developed. Those without stroke developed would follow up until the end of 2013 or the date withdrew from the study. Stroke type was grouped into four groups: HS (ICD-9 codes 430–432), IS (ICD-9 code 434), TIA (ICD-9 code 435), and other acute and other ill-defined cerebrovascular diseases (OCD, ICD-9 codes 436 and 437).

#### Average Medical Cost and Length of Stay

An average of all medical, OPD, and IPD costs at stroke onset and within 30 days of stroke in the two cohorts was presented. At stroke onset, intensive care unit (ICU) care and emergency room (ER) cost and length of stay were also presented.

### Data Analysis

The incidence of the overall stroke and stroke subtype was calculated in two cohorts: the sum of stroke developed divided by the sum of follow-up year (per 1,000 person-years). Cox proportional hazard regression was used to estimate dysmenorrhea cohort to comparison cohort hazard ratio (HR) of stroke, and age-specific proportional distributions of stroke were estimated by stroke subtype. The chi-square test was used to test the different subtype strokes between two cohorts, and the Cochran–Armitage trend test was used to test the proportion of stroke subtype with age. We used logistic regression analysis to calculate the OR and 95% CI of hospitalization by the subtype of stroke for the dysmenorrhea cohort relative to comparisons in those with stroke development. The average medical costs by the subtype of stroke were compared between the two cohorts by linear regression after adjusting for age. The *t*-test was used to examine the different medical costs for stroke and length of stay between the two cohorts.

## Results

### Stroke Development and Stroke Hospitalization Risk

Both cohorts were followed until the end of 2013 to identify cases with incident stroke: 585 (0.89%) cases in the dysmenorrhea cohort and 448 (0.68%) cases in the comparison cohort without dysmenorrhea.

All 1,033 stroke women developed stroke, 585 from the dysmenorrhea cohort and 448 from the comparison cohort. The incidence of stroke was 1.17 and 0.94 per 1,000 person-years in the dysmenorrhea, and the comparison cohort and corresponding risk were 1.26 (95% CI = 1.11–1.42) after adjusting for age (Table 1). The incidences of each type of stroke in the dysmenorrhea and comparison cohort, respectively, were 0.19 and 0.20 per 1,000 person-years for HS, 0.23 and 0.19 for IS, 0.37 and 0.23 for TIA, and 0.38 and 0.32 for others. Compared to the comparison cohort, the dysmenorrhea cohort was significantly different only in TIA (HR = 1.64, 95% CI = 1.29–2.08).

**Table 1:** Incidence and hazard ratio for stroke between dysmenorrhea by Cox proportional hazard regression after adjusting for age.

	**HS**	**IS**	**TIA**	**Other**	**Total**
Comparison					
Person-years	478,074				
Event no.	95	90	108	155	448
Incidence^†^	0.20	0.19	0.23	0.32	0.94
HR (95% CI)	Ref.	Ref.	Ref.	Ref.	Ref.
Dysmenorrhea					
Person-years	497,961				
Event no.	97	116	183	189	585
Incidence^†^	0.19	0.23	0.37	0.38	1.17
HR (95% CI)	0.98	1.24	1.64	1.18	1.26
	(0.74–1.30)	(0.94–1.63)	(1.29–2.08)	(0.95–1.46)	(1.11–1.42)

† *per 1,000 person-years*.

Women who developed stroke were younger in the dysmenorrhea cohort than in the comparison cohort (Table 2). We noted that the proportion of HS declined with age (*p* for trend <0.0001), while the proportion of IS and TIA increased with age (*p* for trends 0.049 and 0.015). The overall proportion of TIA was higher in the dysmenorrhea cohort than in the comparison cohort (31.3 vs. 24.2%, *p* = 0.01).

**Table 2:** Stroke types by age compared between women with and without dysmenorrhea.

**Dysmenorrhea**	**Stroke type**									
**Age, years**	**HS**		**IS**		**TIA**		**Other**		**Total**	
	***n***	**%**	***n***	**%**	***n***	**%**	***N***	**%**	***N***	**%**
Yes										
Total	97	16.6	116	19.8	183	31.3	189	32.3	585	100.0
15–24	43	34.1	19	15.1	28	22.2	36	28.6	126	21.5
25–34	20	12.7	29	18.4	56	35.4	53	33.5	158	27.0
35–44	34	11.3	68	22.6	99	32.9	100	33.2	301	51.5
*p* for trend^a^	<0.0001		0.064		0.070		0.410			
No										
Total	95	21.3	90	20.1	108	24.2	155	34.5	448	100.0
15–24	30	36.1	13	15.7	15	18.1	25	30.1	83	18.5
25–34	24	19.1	27	21.4	28	22.2	47	37.3	126	28.1
35–44	41	17.2	50	20.9	65	27.2	83	34.7	239	53.4
*p* for trend^a^	0.001		0.388		0.077		0.605			
*p* for trend^a^^,^ ^b^	<0.0001		0.049		0.015		0.325			
*p*-value^c^	0.06		0.92		0.01		0.44			

*HS, hemorrhagic stroke; IS, ischemic stroke; TIA, transient cerebral ischemia; Other, other acute and other ill-defined cerebrovascular diseases*.

a *Cochran–Armitage trend test*.

b *To test the trend with age in all study subjects*.

c *Compared patients with dysmenorrhea and comparisons using chi-square test*.

Table 3 compares the risk of hospitalization by stroke subtype in those women with stroke development. Compared to the comparison cohort, the dysmenorrhea cohort showed a significantly lower risk for IS (adjusted OR = 0.48, 95% CI = 0.21–0.69).

**Table 3:** Estimated risk of hospitalization for stroke in women with dysmenorrhea compared to women without dysmenorrhea by the subtype of stroke.

		**Hospitalization**		**OR (95% CI)**	
**Stroke type**	**Cohort**	**Yes**	**No**	**Crude**	**Adjusted**
HS	Dysmenorrhea	51	46	1.23 (0.70–2.17)	1.26 (0.71–2.23)
	Comparison	45	50	Reference	Reference
IS	Dysmenorrhea	27	89	0.38 (0.21–0.69)	0.48 (0.21–0.69)
	Comparison	40	50	Reference	Reference
TIA	Dysmenorrhea	16	167	0.70 (0.32–1.52)	0.69 (0.32–1.50)
	Comparison	13	95	Reference	Reference
Other	Dysmenorrhea	11	178	1.31 (0.49–3.46)	1.30 (0.49–3.44)
	Comparison	7	148	Reference	Reference

*HS, hemorrhagic stroke; IS, ischemic stroke; TIA, transient cerebral ischemia; Other, other acute and other ill-defined cerebrovascular diseases*.

*Use comparison as reference; adjusted CI, controlling for age*.

### Medical Cost for Stroke and Length of Stay

Table 4 shows the average costs of stroke by stroke subtype. The mean costs were generally lower in the dysmenorrhea cohort than those in the comparisons; none of the differences were significant. The average HS cost to IS cost ratios were nearly 4.1 in women with dysmenorrhea (US$ 3254/789) and 3.3 in comparisons (US$ 3870/1171). The cost of care for TIA was the least. The overall average cost for stroke cases was thus moderately lower in the dysmenorrhea cohort.

**Table 4:** Comparison of the average cost of stroke care (USD) between women with and without dysmenorrhea by stroke subtype.

	**Dysmenorrhea**							
	**Yes**			**No**				
**Stroke type**	***N* (%)**	**Mean**	**SD**	***N* (%)**	**Mean**	**SD**	**Difference**	***P***
HS	97 (16.6)	3254	4890	95 (21.2)	3870	8000	−616	0.52
IS	116 (19.8)	789	2382	90 (20.2)	1171	2924	−382	0.30
TIA	183 (31.3)	157	254	108 (24.1)	183	267	−26	0.41
Other	189 (32.3)	255	954	155 (34.6)	324	1426	−69	0.59
Total	585 (100)	827	2566	448 (100)	1212	4232	−385	0.07

*HS, hemorrhagic stroke; IS, ischemic stroke; TIA, transient cerebral ischemia; Other, other acute and other ill-defined cerebrovascular diseases; SD, standard deviation*.

The dysmenorrhea cohort was less likely to be hospitalized (18.0 vs. 23.4%, *p* = 0.03) and cared at ICU (7.86 vs. 11.6%, *p* = 0.04). Mean costs for hospitalization cares, ICU cares, emergency cares, and outpatient cares were all less for the dysmenorrhea cohort than for the nondysmenorrhea cohort but not at a significant level (Table 5).

**Table 5:** Cost of hospitalization, uses of intensive care unit, emergency room, and outpatient visit for patients with stroke compared between women with and without dysmenorrhea.

	**Dysmenorrhea**		
	**Yes**	**No**	***p*-value**
	***N* = 585**	***N* = 448**	
Hospitalization (included ICU)			
Yes, *n* (%)	105 (18.0)	105 (23.4)	0.03^a^
Mean days ± SD	19.9 ± 27.5	20.2 ± 34.2	0.94^b^
Mean cost ± SD	3822.5 ± 4817.0	4557.0 ± 7716.9	0.41^b^
ICU care received			
Yes, *n* (%)	46 (7.86)	52 (11.6)	0.04^a^
Mean cost ± SD	1680.3 ± 1396.3	2648.9 ± 3279.0	0.06^b^
OPD (included ER)			
Mean cost ± SD	148.7 ± 155.2	157.3 ± 190.9	0.46^b^
ER			
Mean cost ± SD	300.5 ± 215.3	326.9 ± 250.8	0.42^b^

*SD, standard deviation; ICU, intensive care unit; OPD, outpatient department; ER, emergency room*.

*n, number of persons hospitalized in each group. Mean days and SD are calculated only for those hospitalized*.

a *Chi-square test*.

b *t-test*.

**Figure 2:**
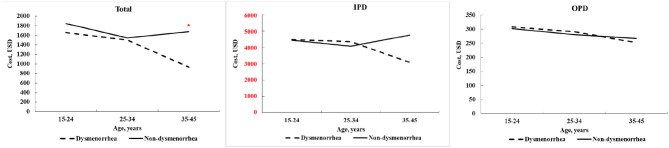
Average costs of stroke care estimated within 30 days post stroke compared by age between women with and without dysmenorrhea.

Figure 2 shows the trends on the average costs of stroke care by age between the dysmenorrhea cohort and comparisons within 30 days post stroke. We noted that the average total costs for the stroke care were similar for women with dysmenorrhea and without dysmenorrhea in women younger than 35 years but with a greater gap between the 35- and 44-year groups (*p* = 0.02).

## Discussion

Our study compared strokes between young women with and without dysmenorrhea, focusing on the costs of stroke care by the patient age and stroke subtype, and found that subtypes differed by age and costs differed by subtype and age. The distribution of stroke and costs for stroke care may vary by population, sex, age, and other factors such as comorbidity (24, 25). We found that HS cost of stroke care is far more (US$3254) than ischemic stroke (US$2382), and this difference is −616. Our study provided the much-needed economic information for evaluating the cost-effectiveness of programs for managing or preventing stroke. A study in the United States found that the hospitalization cost for HS is higher than that in IS. Moreover, HS requires more complicated medical treatment (26). An early study in Taiwan found that the average hospitalization cost for stroke decreases with age, and the average hospitalization cost for children is 1.8 times that of adults. This is because the incidence of HS in children is more than three times higher than in adults (25). Additionally, HS requires more complicated medical treatment. Our study has similar results, that is, the cost of HS is the highest among all types of stroke patterns in young women, regardless of having dysmenorrhea.

Compared with the nondysmenorrhea cohort, women with a dysmenorrhea history had a higher proportion of TIA but lower proportions of HS, IS, and OCD. TIA is a transient ministroke, self-resolving, and causing no permanent impairment (27, 28). The cost of TIA treatment in this study was substantially lower than those of all other types of strokes, with a mean cost of ~20% of the cost of IS treatment or 4.8% of the cost of HS treatment for women with dysmenorrhea. The medical cost of TIA is lower than that of other strokes. It is because most of the signs and symptoms of TIAs disappear within an hour, though rarely symptoms may last up to 24 h before a stroke. Most of the patients seek medical attention immediately before a stroke. Timely assessment and identification of potentially treatable diseases may help prevent stroke and reduce medical costs.

These differences in treatment costs between TIA and IS, or HS could also be seen in women in the comparison group. A Malaysian study also observed higher costs for HS care (29). It is worth noting that the average costs of caring for four types of stroke were not significantly different between the two cohorts.

However, our data showed that women with dysmenorrhea have a lower hospitalization rate and a lower ICU care rate for stroke than the comparison group. A lower hospitalization and ICU care rates may explain the lower overall cost for stroke care in women with dysmenorrhea. We speculate that it may be because they received more frequent medical attention for their dysmenorrhea or other disorders. To support this claim, we further conducted a *t*-test to compare the number of outpatient visits between two cohorts before they received the diagnosis of stroke and found that the average number of outpatient visits in the dysmenorrhea group (16.7 ± 13.6) was significantly higher than that of the nondysmenorrhea group (11.3 ± 11.0; *p* < 0.0001). The result of the analysis could be supported by the lower cost of the dysmenorrhea cohort in the long run. Nevertheless, the relationship among dysmenorrhea, the number of visits, and disease severity still needs more evidence.

Among outpatients, we reported that no obvious age-dependent difference in costs existed between the two cohorts. We also failed to observe the difference in the total costs of stroke care for younger women between the two cohorts. However, the difference in inpatient costs of stroke care existed between women with and without dysmenorrhea for only those aged 35–44 years. The average cost of inpatient care for the nondysmenorrheal cohort peaked in those aged 35–44 years old. This is because the incidence of stroke increases with age. The stroke-related costs including treatment and rehabilitation may be associated with underaged victims. Many investigators have conducted studies of the health and economic burden of this disease among older individuals (25, 26). This explains that the total cost gap between the two cohorts was larger in the 35–44 year group than in the younger group. In the dysmenorrhea cohort, the cost of hospital care and the total cost of the older individuals have decreased, while in contrast, the cost of the youngest group has increased. The cost of stroke care for women without dysmenorrhea seems to be affected by age and stroke type.

A study carried out using the MarketScan Commercial Claims and Encounter inpatient database of 97,374 hospitalizations in the United States also showed a higher cost of stroke care for younger patients than that for older patients (26). The mean cost is 1.7-fold higher for patients with HS stroke than those with IS, and younger patients are more prevalent with HS. However, unlike our study, the MarketScan study considered only two age groups (above and below 45 years) (26). Age is an important factor associated with the proportional distribution among subtypes of stroke. In our study population, HS is more prevalent in younger women than in older women. Higher portions of HS were observed in all ages in comparisons than in the dysmenorrhea cohort.

The findings regarding the hospitalization of patients having stroke with and without dysmenorrhea are unique to this study. Our findings on the costs of stroke care for women with and without dysmenorrhea can be verified in other population research. In this study, we found no age-dependent difference in outpatient care costs between women with and without dysmenorrhea. However, the overall mean cost of stroke care is moderately lower in women with dysmenorrhea. Nonetheless, costs are different among stroke types, which may vary by age since stroke types vary by age. The cost of stroke care for individual women with and without dysmenorrhea is associated with stroke type. Those with HS require more expensive care than those with TIA. More research is required to further explore why the HS risk is greater in younger women since nearly 35% of cases were HS in the 15–24 years old. It is also important to conduct further research to determine the factors apart from age and stroke type that influence the cost of stroke care. Based on our findings, strategies need to be put in place to help younger women in both cohorts and older women in comparisons, particularly for those suffering from HS.

## Conclusions

The average cost for stroke care was not significantly different between women with and without dysmenorrhea. Since the hospitalization rate and medical costs of TIA are lower than other types, early prevention and treatment are recommended for women with or without dysmenorrhea who develop TIA to avoid recurrence or progression to other types of stroke and reduce medical costs.

### Strengths and Study Limitations

In general, stroke is relatively rare in the younger population. The present study is strengthened by using large population-based data to compare stroke risk in women aged 15–44 with and without dysmenorrhea. The findings regarding the hospitalization of patients having stroke with and without dysmenorrhea are particularly unique to this study.

Despite this being the first study to compare the risk of stroke and cost for care between women with and without dysmenorrhea by stroke subtype, it should be noted that our study does not detail the factors associated with hospitalization among patients with stroke. Hence, it is important for future researchers to further assess the factors associated with hospitalization among patients with stroke in addition to stroke subtype. We recommend a focus on stroke-related comorbidity and sociodemographic status of the study population. Another study limitation required to address is that women with minor aches during menstruation were likely not included in the dysmenorrhea cohort. Some of these women might be selected into the comparison cohort. Furthermore, information on lifestyle, such as drinking and smoking, was unavailable in the claims data. The impact was likely not existing because few women in Taiwan drink or smoke (30).

The final limitation is that dysmenorrhea included primary dysmenorrhea and secondary dysmenorrhea due to the ICD-9 code diagnosis; we cannot distinguish between primary dysmenorrhea and secondary dysmenorrhea. We were unable to identify the two types to evaluate the attribution to the medical costs further.

## Data Availability Statement

The datasets presented in this article are not readily available because the data were obtained from the database of the Taiwan National Health Research Institutes. The authors are not eligible to duplicate and disseminate the database. For further access to the database, please contact the Ministry of Health and Welfare (Email: stcarolwu@mohw.gov.tw) for assistance. Taiwan Ministry of Health and Welfare Address: No. 488, Sec. 6, Zhongxiao E. Rd., Nangang Dist., Taipei City 115, Taiwan (R.O.C.). Phone: +886-2-8590-6848). Requests to access the datasets should be directed to stcarolwu@mohw.gov.tw.

## Ethics Statement

The study was approved by The Research Ethics Committee of China Medical University and Hospital (CMUH104-REC2-115-AR4). Written informed consent for participants was not required for this study in accordance with the national legislation and the institutional requirements.

## Author Contributions

Y-WL and M-HL: conceptualization and writing (original draft). C-HM and F-CS: data curation. Y-LT, C-HK, and Y-WL: validation. C-HK and Y-LT: resources and supervision. All authors: investigation, methodology, writing, reviewing, and editing.

## Conflict of Interest

The authors declare that the research was conducted in the absence of any commercial or financial relationships that could be construed as a potential conflict of interest.

## Publisher's Note

All claims expressed in this article are solely those of the authors and do not necessarily represent those of their affiliated organizations, or those of the publisher, the editors and the reviewers. Any product that may be evaluated in this article, or claim that may be made by its manufacturer, is not guaranteed or endorsed by the publisher.
